# Editorial

**DOI:** 10.1120/jacmp.v1i2.2644

**Published:** 2000-03-01

## Editorial

I thank Editor Peter Almond for inviting me to write this Editorial for the second issue of the Journal of Applied Clinical Medical Physics (JACMP). This gives me the opportunity to be one of the first to congratulate Professor Almond, his Advisory and Editorial Boards, and the American College of Medical Physics (ACMP) on their outstanding new contribution. I especially want to recognize the contributions of Associate Editor Mike Mills. It was Mike who first thought of this journal several years ago, and without Mike's foresight, enthusiasm, and persistence, it is unlikely that this journal would have made it to our desktops today.

What impresses me most about the JACMP is its professional, polished appearance. You would never know it is a brand new journal. Much of this is due to the inspired selection of the AIP as the publisher. The American Institute of Physics (AIP) has considerable experience with putting journals online, including our other journal, Medical Physics. I am especially pleased that the formats of our two online journals are so similar. There is considerable overlap in our readership so it is appropriate that readers who become familiar with maneuvering through one journal will find it seamless (and painless) to move through the other. When the JACMP was first proposed, many of us feared that it might be envisioned as a rival to Medical Physics. The last thing we needed was another scientific journal of medical physics. With this in mind, it was decided that the JACMP would concentrate on publication of clinical, professional, and educational articles, as opposed to the research manuscripts and extensive reports published in Medical Physics. In my mind, there is no question: the JACMP complements rather than competes with Medical Physics. Indeed, for the past six months‐or‐so, the Associate Editors of Medical Physics and I have frequently recommended that some of the manuscripts submitted to Medical Physics would be better off published in the JACMP. This is often done before extensive review, which would otherwise delay submission to the JACMP. Our new journal is clearly of benefit to authors and readers alike and, as an Editor, I can attest that it is also of benefit to me. I have often felt dejected because I have had to inform an author of the rejection of a well‐written article just because it was out of the scope of Medical Physics, especially when there was no suitable alternative journal to which to refer it. This is no longer the case.

I am especially interested in the fully online nature of this journal: online submission, online review, and online publication. I have no doubt that this is the way journal publications will have to go in the near future. Indeed, I am already watching the progress of the JACMP with the intention of using many of the online features for Medical Physics.

Congratulations Peter on an excellent start to an outstanding journal. With your leadership, I am confident that the JACMP will soon become a major player in the medical physics publication arena.

Colin G. Orton, Ph.D.

Editor, Medical Physics

